# Stacking Engineering of Heterojunctions in Half‐Metallic Carbon Nitride for Efficient CO_2_ Photoreduction

**DOI:** 10.1002/advs.202307192

**Published:** 2023-12-10

**Authors:** Xingwang Zhu, Hangmin Xu, Jinyuan Liu, Chuanzhou Bi, Jianfeng Tian, Kang Zhong, Bin Wang, Penghui Ding, Xiaozhi Wang, Paul K. Chu, Hui Xu, Jianning Ding

**Affiliations:** ^1^ College of Environmental Science and Engineering, Institute of Technology for Carbon Neutralization Yangzhou University Yangzhou 225009 P. R. China; ^2^ Department of Physics, Department of Materials Science and Engineering, and Department of Biomedical Engineering City University of Hong Kong Tat Chee Avenue Kowloon Hong Kong 999077 P. R. China; ^3^ School of the Environment and Safety Engineering, Institute for Energy Research Jiangsu University Zhenjiang 212013 P. R. China; ^4^ Department of Science and Technology Linköping University Norrköping SE‐601 74 Sweden

**Keywords:** BiOBr, CO_2_ reduction, half‐metallic C(CN)3, heterojunctions, photocatalysis

## Abstract

Enhancing charge separation in semiconductor photocatalysts is a major challenge for efficient artificial photosynthesis. Herein, a compact heterojunction is designed by embedding half‐metallic C(CN)_3_ (hm‐CN) hydrothermally in BiOBr (BOB) as the backbone. The interface between hm‐CN and BOB is seamless and formed by covalent bonding to facilitate the transmission of photoinduced electrons from BOB to hm‐CN. The transient photocurrents and electrochemical impedance spectra reveal that the modified composite catalyst exhibits a larger electron transfer rate. The photocatalytic activity of hm‐CN/BOB increases significantly as indicated by a CO yield that is about four times higher than that of individual components. Density‐functional theory calculations verify that the heterojunction improves electron transport and decreases the reaction energy barrier, thus promoting the overall photocatalytic CO_2_ conversion efficiency. The half‐metal nitride coupled semiconductor heterojunctions might have large potential in artificial photosynthesis and related applications.

## Graphical abstract

A unique synthesis method is utilized to establish a Z‐Scheme heterojunction with close contact between BiOBr and half‐metallic C(CN)_3_. The efficient electron transfer at the interface is improved through this compact structure, which enhances the material's redox capacity and facilitates the photocatalytic CO_2_ reduction.

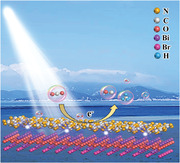

## 1 Introduction

Photocatalytic CO_2_ reduction is an advanced technology for converting CO_2_ into fuels such as CO, HCOOH, CH_3_OH, and CH_4_.^[^ ^1^, ^2^, ^3^ ^]^ However, CO_2_ is a highly stable molecule (ΔG^θ^ = −394 kJ mol^−1^) with a large carbon‐oxygen double bond energy (≈803 kJ mol^−1^).^[^ ^4^, ^5^, ^6^ ^]^ Therefore, photocatalysts for the reaction require more negative reduction potentials and efficient electron transfer to harness the CO_2_ resource. Despite recent advances in strategies such as ion doping, porosity modulation, cocatalysis, and defect engineering, the CO_2_ conversion rate still cannot meet commercial requirements.^[^ ^7^, ^8^, ^9^, ^10^ ^]^ This is because the intrinsic CO_2_ capture capability of most photocatalysts is limited.^[^ ^11^, ^12^ ^]^ For example, some semiconductors with moderate CO_2_ adsorption activity have poor electrical conductivity. Although narrow‐bandgap semiconductors promote visible light absorption, this contradicts the wide bandgap required for simultaneous CO_2_ conversion, as a negative conduction band is needed to meet the thermodynamic requirements for CO_2_ reduction.^[^ ^1^, ^13^ ^]^ Furthermore, the fast carrier recombination and slow interfacial charge transport impede the participation of electrons and holes in the reaction and reduce the solar conversion efficiency.

The photocatalytic CO_2_ conversion efficiency of pristine bismuth‐based photocatalysts is constrained by the low conduction band position, suboptimal photoinduced charge transfer efficiency, as well as sluggish surface reaction kinetics.^[^ ^14^, ^15^ ^]^ To overcome these limitations, heterojunctions composed of photoactive materials can constrain the energy levels at the edges of the conduction and valence bands of the semiconductor. Moreover, electrons and holes generated by light are transferred to two or more components forming the heterojunction interface. This process suppresses recombination and improves the photocatalysis activity^[^ ^16^, ^17^ ^]^ and consequently, the heterojunction strategy inspired by natural photosynthesis has gained widespread attention. Excited electrons are more likely to transfer from the semiconductor with a smaller work function (WF) to the semiconductor with a larger WF to form an internal electric field in the semiconductor.^[^ ^18^, ^19^ ^]^ The Z‐scheme heterojunctions consume some of the photogenerated electrons and holes through the internal electric field generated, so that the electrons and holes with stronger redox capabilities are separated and used in photocatalytic reactions.^[^ ^20^ ^]^ Therefore, combining a high‐quality reducing semiconductor with an oxidizing semiconductor is desirable for artificial photosynthesis. For instance, the photocatalytic CO_2_ reduction activity of BiOBr (BOB) can be boosted by the incorporation of gold.^[^ ^21^ ^]^ Additionally, improving the performance of BOBphotocatalytic reduction of CO_2_ can be accomplished through the application of precious metal loading. However, considering the high cost and natural scarcity of noble metals, utilizing a low‐cost and readily available cocatalyst instead of noble metal is particularly important.

Half‐metallic C(CN)_3_ (hm‐CN) is a unique organic semiconductor that can be prepared by thermal condensation using nitrile‐functionalized imidazolium ionic liquids.^[^ ^22^, ^23^ ^]^ The hm‐CN framework consists of carbon atoms that connect the triazine rings and spin‐polarized carbon atoms, resulting in good charge transfer to the non‐metallic materials.^[^ ^23^, ^24^ ^]^ The high photocatalytic activity of hm‐CN is attributed to effective electron‐hole separation facilitated by the transition from the singlet to triplet state besides favorable charge transfer. Moreover, the electron‐rich nature and abundance of nitrogen atoms in hm‐CN enable it to exhibit strong CO_2_ adsorption.^[^ ^25^ ^]^ For example, Zhou and Yang have incorporated hm‐CN into graphitic carbon nitride (g‐C_3_N_4_) to enhance CO_2_ capture and conversion.^[^ ^22^, ^23^ ^]^ As a cocatalyst, hm‐CN outperforms Pt, yielding a CO output that is 1.9 times greater than that of g‐C_3_N_4_ modified with Pt. Shen et al. have developed a composite catalyst by combining hm‐CN with BiVO_4_ to construct the heterojunction.^[^ ^24^ ^]^ The optimized BiVO_4_/hm‐CN‐20 catalyst possesses a layered structure with ample exposed active sites and enhanced electron capture cross‐sections for selective reduction of CO_2_ to CO. Because of the half‐metallic properties, hm‐CN not only exhibits specific metallic characteristics such as high electrical conductivity, catalytic kinetics, small energy barriers, and good carrier transport, but also is cheaper than noble metals. It is worthwhile to investigate how to utilize the benefits of hm‐CN in conjunction with other semiconductors to enhance the activity of photocatalytic reduction of CO_2_.

Herein, a solar photosynthesis system incorporating half‐metallic carbon nitride [hm‐CN] nanosheets coupled with BiOBr is designed and demonstrated. The materials with a negatively charged surface are synthesized with a cationic ionic liquid with thetricyanomethanide anion, and hm‐CN is subsequently combined with BOB (hm‐CNB) to form a layered photocatalyst with abundant heterojunction interfaces and reaction sites. The stability of the interface was reinforced by a one‐step solvothermal method at high temperature and pressure. The optimized hm‐CNB shows outstanding photocatalytic activity such as a CO production rate of 183.62 µmol g^−1^ h^−1^ and CH_4_ production of 27.88 µmol g^−1^ h^−1^. This work provides guidance for the design of high‐performance photocatalysts suitable for use in the field of photoreduction CO_2_.

## 2 Synthesis

### 2.1 Chemicals

Bi(NO_3_)_3_·5H_2_O, NaBr, ethanol (EtOH), ethylene glycol (EG), C_4_N_3_Na, C_8_H_15_N_2_Br, and C_4_H_8_O_2_ were bought from Shanghai Sinopharm and used without purification. Deionized water was used in the preparation and measurement.

### 2.2 Preparation of Bulk g‐C_3_N_4_ (CN) and BiOBr (BOB)

Urea (10 g) was heated in a muffle furnace at 550°C at a rate of 5 °C min^−1^ for 4 h and after cooling, the yellow powder was bulk g‐C_3_N_4_ (CN). Bi(NO_3_)_3_·5H_2_O (0.1 mmol) and NaBr (0.1 mmol) were dissolved in EG (5 mL) and stirred for 30 min to obtain solution A. EtOH (30 mL) was added to solution A and stirred for 30 min to form solution B. Solution B was reacted at 160°C for 24 h in a Teflon‐lined reactor (50 mL). The resulting product was washed with deionized water and EtOH and vacuum‐dried at 60°C for 8 h. The sample was denoted as BiOBr (BOB).

### 2.3 Preparation of [BMIm][C(CN)_3_]

C_4_N_3_Na (0.01 mol) and C_8_H_15_N_2_Br (0.01 mol) were added to 150 mL of deionized water (150 mL), stirred for 12 h, and extracted with ethyl acetate. The organic and aqueous phases were separated and the organic phase was retained and washed with deionized water to remove bromine salts. It was placed in a rotary evaporator and heated to 60°C to remove ethyl acetate at a rotating speed of 100 rpm min^−1^ to obtain a light‐yellow oil designated as [BMIm][C(CN)_3_].

### 2.4 Synthesis of Half‐Metallic C(CN)_3_ (hm‐CN) and hm‐CN/BiOBr (hm‐CNB)

The [BMIm][C(CN)_3_] oil‐like product was laid flat on a ceramic ark and wrapped in tin foil to prevent from moving down. It was placed in the center of a quartz glass tube after high‐temperature calcination, in which thermal condensation results in reconstitution. In the N_2_ atmosphere, the temperature was increased from room temperature to 400°C at a rate of 5 °C min^−1^ and kept for 1 h to produce black half‐metallic C(CN)_3_ (hm‐CN) with a bright metallic luster. The hm‐CN was weighed and placed in a ball mill jar for the reaction to produce the black powder. A one‐step solvothermal method was used for the preparation of hm‐CNB. 5, 15, and 25 mg of hm‐CN powder were added to solution A in section 2.2, followed by the addition of 30 mL EtOH. After stirring the mixture for 30 min, it was transferred to a 50 mL autoclave and subjected to a reaction at 160°C for 24 h. The obtained product was washed with deionized water and ethanol, and then dried under vacuum at 60°C for 8 h. Depending on the mass of hm‐CN (5, 15, and 25 mg), the samples were denoted hm‐CNB‐1, hm‐CNB‐2, and hm‐CNB‐3, respectively.

### 2.5 Synthesis of Bulk g‐C_3_N_4_/BiOBr (CNB)

The bulk g‐C_3_N_4_/BiOBr (CNB) synthesis followed the same procedure as hm‐CNB, with the inclusion of only 15 mg of bulk g‐C_3_N_4_ to solution A in section 2.2, and other operations were consistent.

## 3 Results and Discussion

### 3.1 Theoretical Calculation

**Figure 1:**
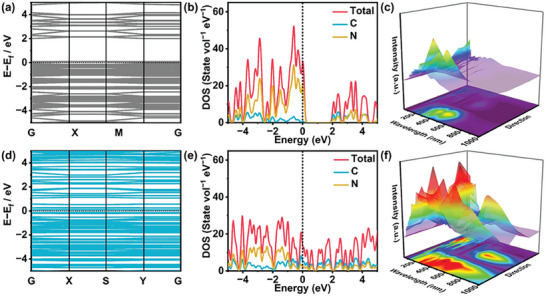
Electronic structures. Band structures of a) CN and d) hm‐CN slab; DOS of b) CN and e) hm‐CN slab with the dashed lines denoting the Fermi levels set at 0 eV; Calculated absorption spectra in different directions of c) CN and f) hm‐CN slab.

To assess the efficacy of the heterojunction system in enhancing the separation of photogenerated electron‐hole pairs and increasing the redox capacity between BiOBr (BOB) and half‐metallic C(CN)_3_ (hm‐CN), various models of BOB, bulk g‐C_3_N_4_/BiOBr (CNB), hm‐CN, hm‐CN/BiOBr (hm‐CNB), and bulk g‐C_3_N_4_ (CN) are developed (Figure S1, Supporting Information). Density‐functional theory (DFT) calculations are performed to analyze the impact on the electronic structure. The density of states (DOS) and band structures of CN and hm‐CN are determined to determine the bandgaps. The bandgap of hm‐CN is narrower than that of CN, implying that electrons in hm‐CN are more readily excited (**Figure** **1**a and d). The DOS of hm‐CN crosses the Fermi level leading to better conductivity and metallic properties^[^ ^26^, ^27^ ^]^ (Figure 1b and e). When CN and hm‐CN are combined with BOB, hm‐CNB and CNB have more pronounced metallic properties including better electrical conductivity and charge capture capability.^[^ ^28^, ^29^ ^]^

**Figure 2:**
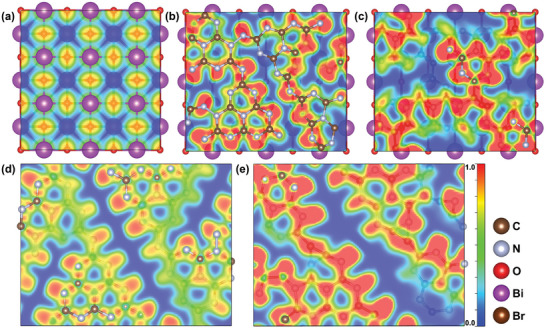
Surface charge density of the models. ELF of a) BOB, b) CNB, c) hm‐CNB, d) CN and e) hm‐CN slab.

The charge density of hm‐CNB is slightly superior to that of CNB, suggesting better semiconducting properties and electron transfer^[^ ^30^ ^]^ because of the heterojunction between hm‐CN and BOB (Figure S2, Supporting Information). The work function of hm‐CN is calculated to be 4.54 eV, which is higher than that of CN (4.36 eV), leading to a lower Fermi level (Figure S3, Supporting Information). Moreover, hm‐CN displays broader absorption than CN and the range can be expanded further by forming a heterojunction with BOB (Figure 1c,f and Figure S4, Supporting Information). The energy loss spectra (Figure S5, Supporting Information) reveal a greater potential for electron spillover from hm‐CNB indicating the contribution of the heterojunction to electron transfer between hm‐CN and BOB.^[^ ^31^, ^32^, ^33^ ^]^ The electronic localization functions (ELF) of the five models are studied and as shown in **Figure** **2**a,d, and e, hm‐CN has a larger surface charge density than CN and BOB. However, the surface charge density of the composites is higher than that of the individual components, with hm‐CNB showing the highest surface charge density. Hence, more electrons can escape from the surface of hm‐CNB to supply more electrons for the adsorption and activation of CO_2_ molecules (Figure 2b,c).

### 3.2 Structure and Composition

The preparation of the hm‐CNB composite catalyst is illustrated in Figure S6 (Supporting Information). The BOB nanosheets are synthesized by the coprecipitation reaction of Bi(NO_3_)_3_ and NaBr. At the same time, the precursor [BMIm][C(CN)_3_] undergoes thermal condensation to produce the half‐metallic carbon nitride hm‐CN which adsorbs onto the BOB nanosheets that are evenly distributed on the hm‐CN surface with the aid of ethylene glycol.

**Figure 3:**
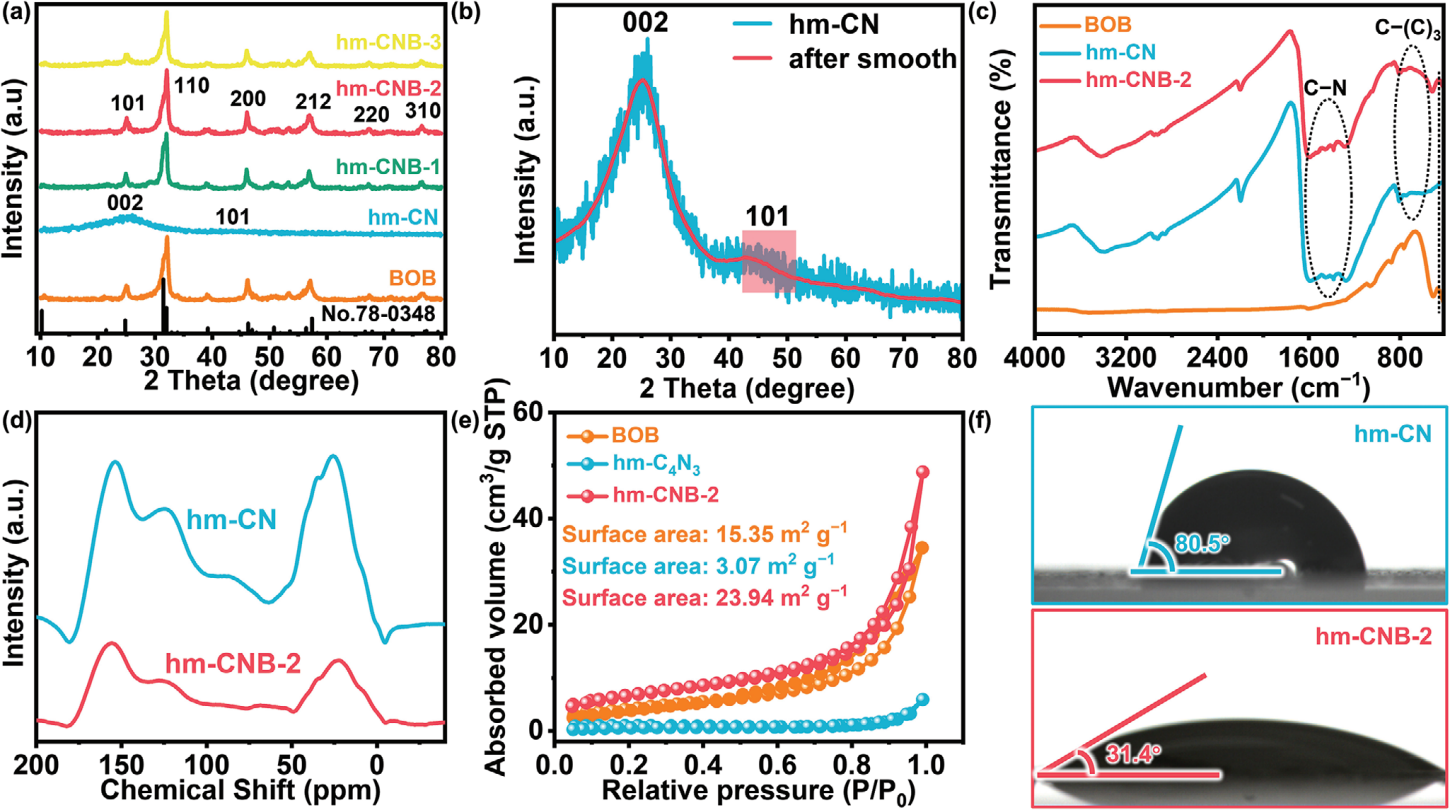
a) XRD patterns of BOB, hm‐CN, and hm‐CNB; b) Magnified XRD patterns of hm‐CN; c) FTIR spectra of BOB, hm‐CN, and hm‐CNB‐2; d) NMR spectra of hm‐CN and hm‐CNB‐2; e) N_2_ adsorption‐desorption isotherms of BOB, hm‐CN, and hm‐CNB‐2; f) Hydrostatic water contact angles on hm‐CN and hm‐CNB.

X‐ray diffraction (XRD) shows that BOB has the PbFCI‐like tetragonal crystal structure (**Figure** **3**a). The diffraction peaks at 25.05°, 32.05°, 46.05°, 57.04°, 67.33°, and 76.64° correspond to the (101), (110), (200), (212), (220), and (310) crystal planes, which match the standard crystal card (JCPDS No.78‐0348).^[^ ^16^, ^21^ ^]^ hm‐CN exhibits two broad peaks at 26.24° and 44.58° for the (002) and (101) planes, respectively, revealing the interlayer stacking structure and modest crystallinity of the conjugated aromatic system (Figure 3b).^[^ ^23^, ^24^ ^]^ When the proportion of hm‐CN doping is small, the XRD patterns of the three composites resemble that of BOB. The intensity of the peak at 25.05° from hm‐CNB‐3 decreases, indicating the incorporation of the additional hm‐CN precursor during preparation and the presence of hm‐CN in the catalyst.^[^ ^22^, ^24^ ^]^ The infrared spectra of hm‐CN and the constituents confirm hybridization of BOB with hm‐CN. As shown in Figure 3c, hm‐CN shows C‐(C)_3_ stretching at 587—794 cm^−1^ and CN heterocyclic vibrations at 1266—1594 cm^−1^.^[^ ^24^ ^]^ After the incorporation of hm‐CN, the composite shows absorption of CN heterocyclic vibrations, which are absent from BOB. Solid‐state nuclear magnetic resonance (NMR) discloses the close contact between BOB and hm‐CN. As shown in Figure 3d, the signals of hm‐CN at 152.48, 124.75, and 87.34 ppm are from sp^2^‐bonded carbon, sp^2^‐hybridized carbon of residual cyanide, and central carbon atom, respectively.^[^ ^22^ ^]^ The signals in the range of 10—50 ppm correspond to marginal carbon atoms. Subsequently, owing to the combination of hm‐CN with BOB, hm‐CNB‐2 shows similar NMR spectra. However, the signal intensity of hm‐CN decreases due to the larger BOB concentration. The results corroborate the incorporation of both components in the formation of the continuous heterojunction.

Based on the N_2_ adsorption‐desorption isotherms in Figure 3e, hm‐CNB‐2 has a larger specific surface area than BOB alone. Consequently, the composite formed by hm‐CN and BOB also has a large specific surface area boding well for the photocatalytic activity. The surface wetness and effects of the heterojunction on the physical properties of the interface are investigated (Figure 3f). The decrease in contact angles promotes better contact between water and hm‐CNB‐2. In addition, hm‐CNB‐2 effectively absorbs carbon dioxide at the interface of the liquid and gas phases and dissolves it in water (Figure S7, Supporting Information). Its hydrophilicity allows it to continuously obtain protons from the water, promoting its reaction with CO_2_.

**Figure 4:**
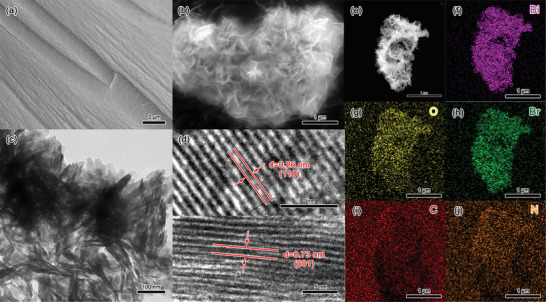
a) SEM image of hm‐CN; b) SEM image, c) TEM image, d) HR‐TEM image, e) STEM image, and f–j) EDX elemental maps of hm‐CNB‐2.

The morphological changes are monitored by scanning electron microscopy (SEM) and transmission electron microscopy (TEM). BOB possesses a nano‐spherical morphology consisting of nanosheets and the unique three‐dimensional structure facilitates carbon dioxide adsorption and light absorption.^[^ ^34^, ^35^ ^]^ The SEM images of hm‐CN and hm‐CNB‐2 (**Figure** **4a** and b) reveal that hm‐CN consists of micron‐sized blocky particles with a smooth layered structure on the surface.^[^ ^22^, ^23^ ^]^ hm‐CNB‐2 sample prepared by the solvent thermal method has a thin encapsulating layer on the surface of the half‐metallic material and some fusion between BOB and hm‐CN. The results are confirmed by Figure 4c showing that the central interior representing the half‐metallic nitride is surrounded by dark‐colored sheet‐like substances. The HR‐TEM image of hm‐CNB‐2 shows clear lattice stripes with lattice spacings of 0.26 nm and 0.73 nm corresponding to the (110) and (001) planes of BOB, respectively (Figure 4d). Scanning transmission electron microscopy (STEM, Figure 4e) and energy‐dispersive X‐ray spectroscopy (EDX, Figure 4f–j) reveal the presence of Bi, O, Br, C, and N. These findings suggest uniform loading of BOB nanosheets on hm‐CN and hybridization of the two materials at the heterojunction interface.

**Figure 5:**
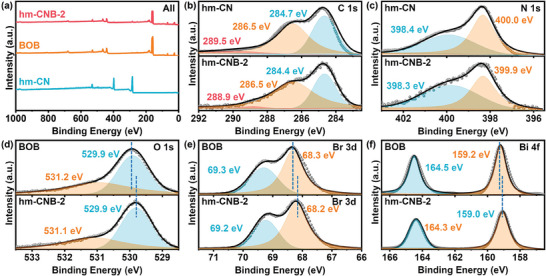
a) Survey XPS spectra of hm‐CN, BOB, and hm‐CNB‐2; b) XPS C 1*s* and c) N 1*s* XPS spectra of hm‐CN and hm‐CNB‐2; d) XPS O 1*s*, e) Br 3*d*, and f) Bi 4*f* spectra of BOB and hm‐CNB‐2.

The X‐ray photoelectron spectroscopy (XPS) spectra of BOB and hm‐CNB in **Figure** **5** show the chemical states of various elements. BOB comprises Bi, O, and Br elements and hm‐CN contains C and N. The XPS survey spectrum (Figure 5a) shows Br, Bi, C, N, and O in line with EDX. To explore the impact of the built‐in electric field, high‐resolution C 1*s* and N 1*s* spectra are acquired from hm‐CN and hm‐CNB‐2 (Figure 5b,c). The C1*s* spectrum of hm‐CN shows sp^2^ C─C bonds (284.7 eV), central carbon (286.5 eV), and sp^2^‐bonded carbon (N─C═N, 289.5 eV).^[^ ^23^, ^36^ ^]^ After addition of BOB, the binding energy of N─C ═N shifts from 289.5 eV to 288.9 eV. The N 1*s* XPS spectrum of hm‐CN (Figure 5c) reveals peaks 398.4 eV and 400.0 eV for C─N═C and C═N, respectively. The two peaks of hm‐CNB‐2 shift to binding energies of 398.3 eV and 399.9 eV, respectively. The O 1*s* spectrum shows peaks at 529.9 eV and 531.2 eV attributed to lattice oxygen and hydroxyl of BOB and hm‐CN, respectively (Figure 5d). The peaks of Br 3*d*_3/2_ (69.2 eV) and Br 3*d*_5/2_ (68.3 eV) in BOB are assigned to Br^−^ as shown in Figure 5e. The Bi 4*f* XPS spectra show peaks of Bi 4*f*_7/2_ (159.2 eV) and Bi 4*f*_5/2_ (164.5 eV) confirming the Bi^3+^ state of BOB (Figure 5f).^[^ ^37^, ^38^ ^]^ The peaks of the same element in hm‐CNB‐2 shift toward lower binding energy, suggesting electron transfer from hm‐CN to BOB due to the heterojunction. The results validate that carbon atoms outside the triazine ring play a significant role in the electronic properties of the materials. The effective charge transfer and strong built‐in electric field between BOB and hm‐CN enhance carrier separation and improve the photocatalytic properties.

### 3.3 Photocatalytic Properties

**Figure 6:**
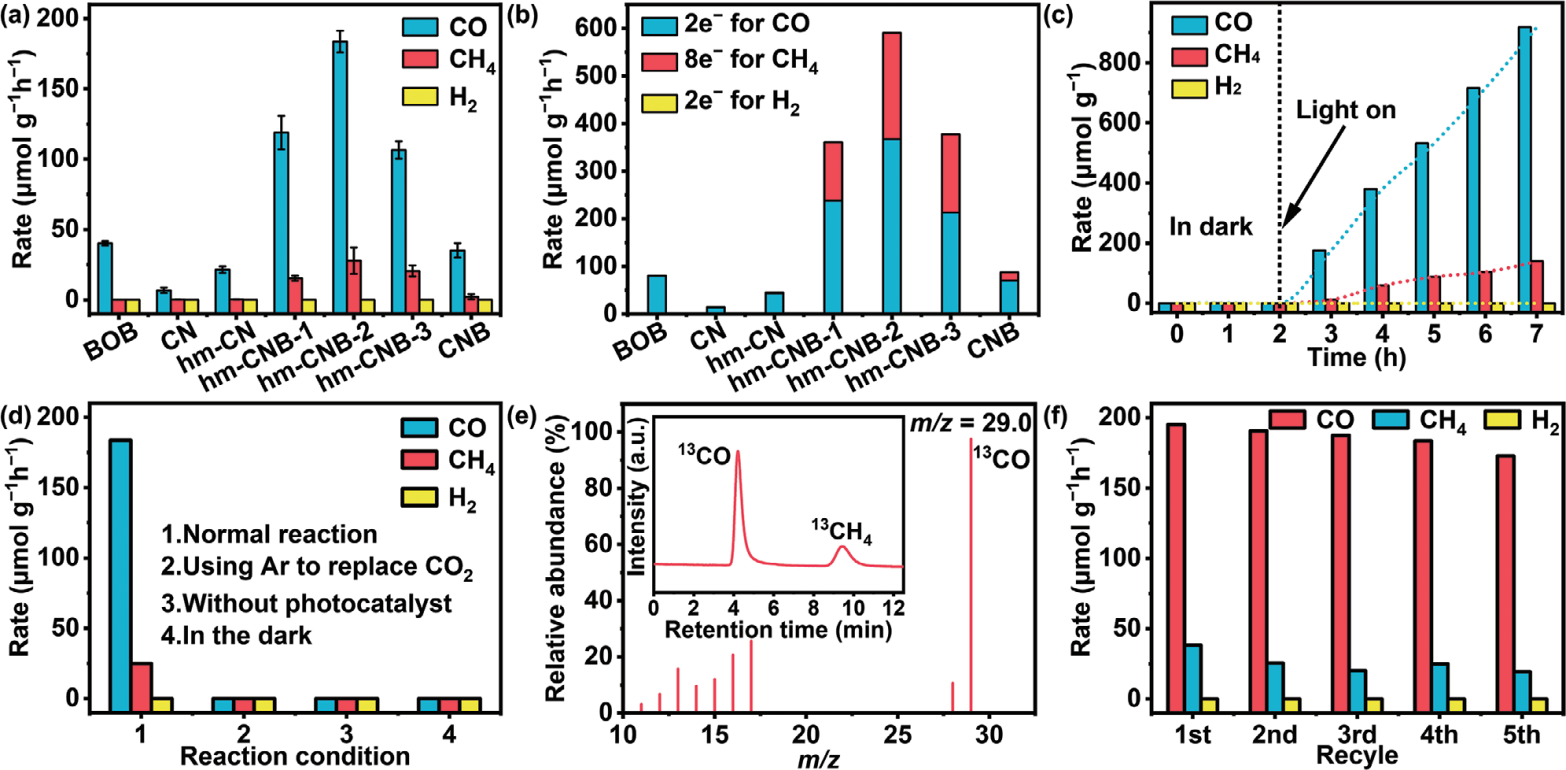
Photocatalytic CO_2_ reduction assessment. a) Comparison of different materials for photocatalytic CO_2_; b) Number of moles of photoinduced electrons in CO_2_ photoreduction; c) Time‐dependent characteristics of hm‐CNB‐2 in photocatalytic CO_2_ reduction; d) CO_2_ reduction of hm‐CNB‐2 under different conditions; e) Mass spectra of the products produced in the photocatalytic reaction of ^13^CO_2_ on hm‐CNB‐2; f) Activity cycles of hm‐CNB‐2.

To simulate photosynthesis by green plants, a sealed liquid‐solid vessel is designed with a xenon lamp. The photocatalytic CO_2_ reduction activities of BOB, CN, hm‐CN, and the hm‐CNB heterojunction are assessed without adding other cocatalysts or photosensitizers. hm‐CN exhibits a higher CO production rate than CN. The photocatalytic CO production rates of BOB and CN are relatively small and they do not produce CH_4_. As for the heterojunction, the activity of CNB is not improved. However, the production rates of both CO and CH_4_ by hm‐CNB increase linearly with hm‐CN concentration and the primary products are CO and a small amount of CH_4_. The optimal activity ratio is observed from hm‐CNB‐2 which shows yield rates of CO and CH_4_ of 183.62 µmol g^−1^ h^−1^ and 27.88 µmol g^−1^ h^−1^, respectively (**Figure** **6a**).

The properties of the composite are compared with those of recently published materials and better photocatalytic CO_2_ reduction characteristics are revealed (Table S1, Supporting Information). By comparing the number of moles of photoexcited electrons generated by the materials, hm‐CNB excited by light produces more electrons, resulting in the largest proportion of moles of photoinduced electrons for CO (Figure 6b). Hence, hm‐CNB generates a substantial quantity of electrons for reduction of CO_2_. To investigate the reaction conditions of CO_2_ reduction, the products of hm‐CNB‐2 under dark and light conditions are compared. When the reaction is conducted without light for 2 h, no product is formed. In contrast, upon light irradiation, the product yield increases (Figure 6c). The control experiments conducted without a light source, without a catalyst, and with Ar replacing CO_2_ reveal no CO or CH_4_ production, suggesting that photocatalytic CO_2_ reduction occurs only under normal reaction conditions (Figure 6d). The carbon sources of the reduction products are identified by ^13^CO_2_ isotopic labeling. As shown in Figure 6e, the peaks at *m/z* of 29 and 17 are ^13^CO and ^13^CH_4_, confirming that the carbon in the product originates from CO_2_.^[^ ^34^, ^39^ ^]^ To assess the stability of hm‐CNB heterojunction, a 25 h cycling test as well as after reaction XRD and TEM tests were performed. The results reveal that the activity decreases slightly after 25 h but the overall stability is good (Figure 6f and Figure S8a,b, Supporting Information).

**Figure 7:**
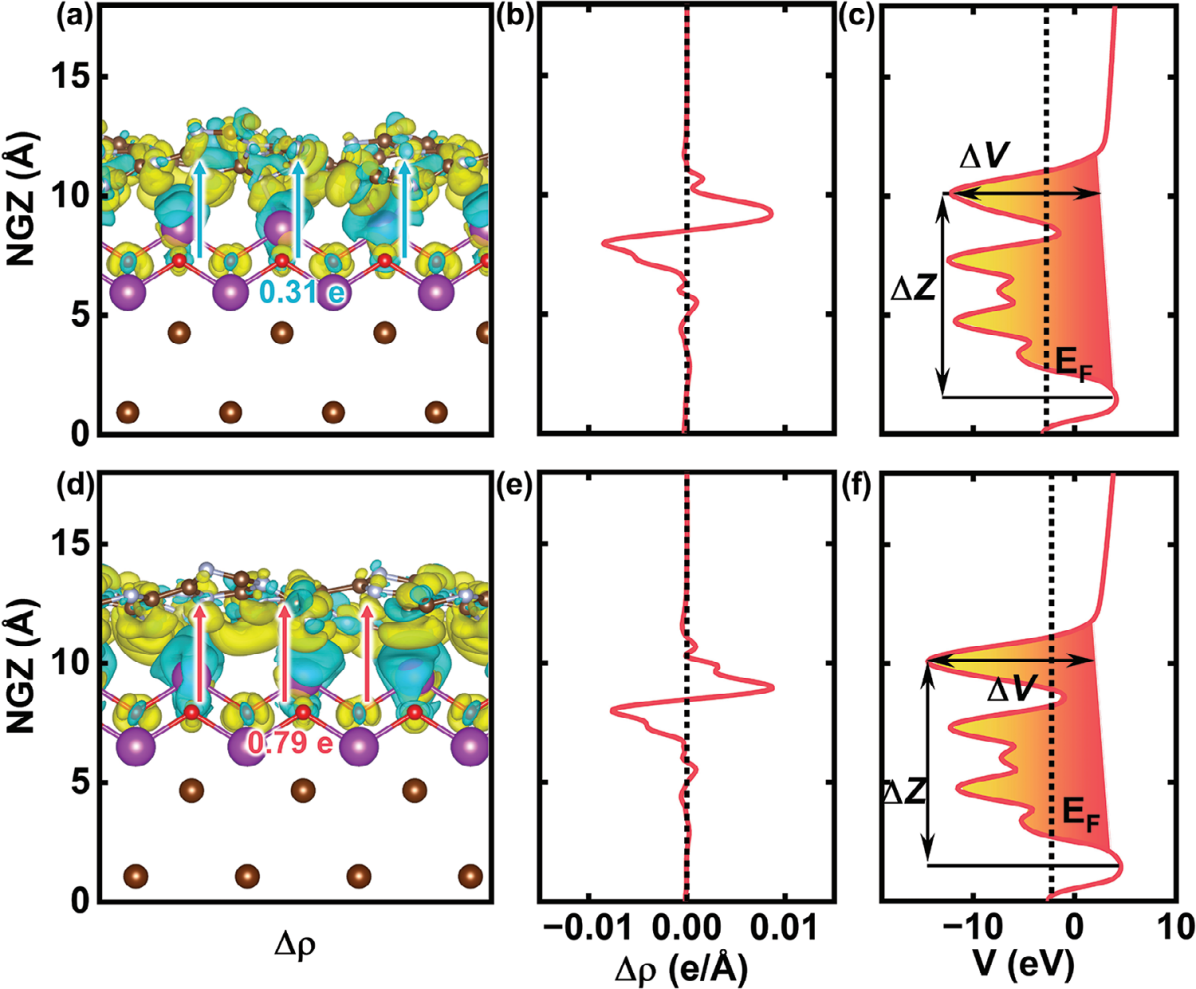
Electronic structure changes in CNB and hm‐CNB. Charge density differences in a) CNB and d) hm‐CNB; b,e) Plane charge density difference and c,f) Plane electrostatic potential along the z‐direction: b,c) CNB and e,f) hm‐CNB.

The mechanism responsible for the enhanced activity of the heterojunction between hm‐CN and BOB is investigated, and the electronic properties of the CNB and hm‐CNB interface are studied theoretically. The difference in the charge density (*Δρ*) is derived by the following equation^[^ ^40^, ^41^ ^]^:

(1) $$\Delta \rho =\rho_{\mathrm{hm}-\mathrm{CNB}}-\rho_{\mathrm{hm}-\mathrm{CN}}-\rho_{\mathrm{BOB}}$$

where ρ_*hm*−*CNB*_, ρ_*hm* − *CN*_, and ρ_*BOB*_ are the total charge densities of hm‐CNB, hm‐CN and BOB, respectively. As shown in **Figure** **7a** and d, hm‐CNB exhibits a smaller charge density redistribution than CNB. Moreover, there is no significant difference in the plane charge density difference along the *z*‐direction between CNB and hm‐CBN (Figure 7b,e). The number of electrons (Δq) transferred between BOB and hm‐CN can be determined using the Bader charge.

(2) $$\Delta q\;\left(z\right)=\int_{-\infty }^{z}\Delta \rho \left(z^{\prime }\right)\;{\mathrm{dz}}^{\prime }$$

Bader charge analysis shows that the in situ synthesized hm‐CNB transfers approximately 0.79 e to the BOB layer, whereas the physically mixed CNB only transfers 0.31 e to the BOB layer. Hence, hm‐CNB has a more robust interface which enhances charge transfer.^[^ ^40^, ^41^ ^]^ The plane‐averaged electrostatic potential along the z‐direction of the interface is derived to examine charge transfer across the interface and the influence of the potential energy barrier (Figure 7c,f). Measured from the bottom of the nearest potential well at the CNB interface, there is a potential drop characterized by ΔV = 16.54 eV and ΔZ = 8.68 Å. In contrast, at the hm‐CNB interface, the tunneling potential shows ΔV = 19.03 eV and ΔZ = 8.68 Å. Remarkably, at the Fermi level, the hm‐CNB tunneling barrier is significantly smaller than the CNB tunneling potential. This phenomenon is expected to facilitate the generation of an internal electric field and to provide a driving force for the separation of photoinduced electrons and holes.^[^ ^32^, ^41^ ^]^

**Figure 8:**
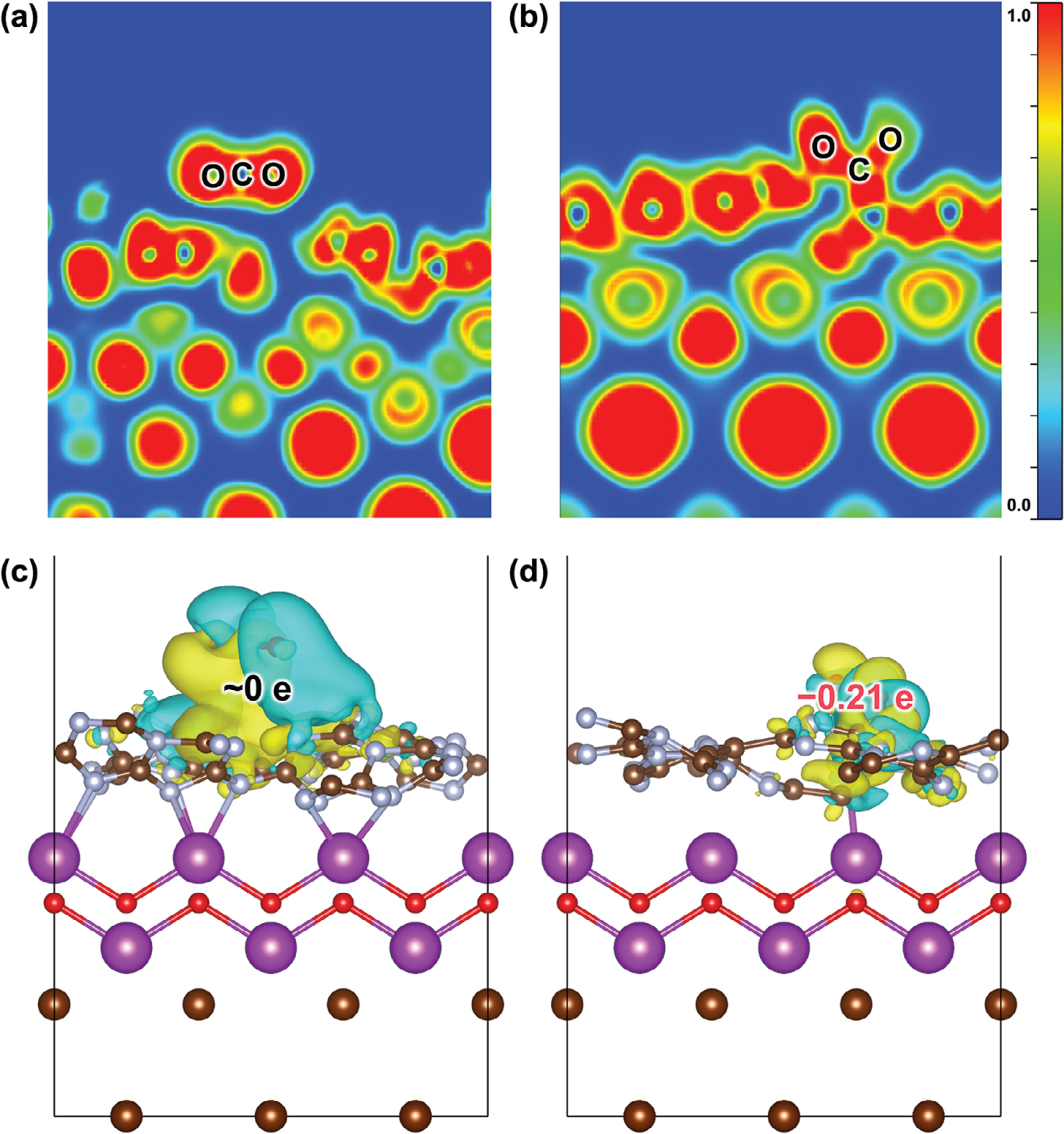
Electronic structures of CNB and hm‐CNB. ELF of a) CNB and b) hm‐CNB, where Δq represents CO_2_ total charge; Differences in the charge density of CO_2_ molecules adsorbed on c) CNB and d) hm‐CNB.

DFT calculations were conducted for CNB and hm‐CNB to gain insights into the enhanced CO_2_ adsorption and charge density difference for electron transport. The ELF indicates more covalent interactions between CO_2_ and the hm‐CNB heterojunction (**Figure** **8a**,b), implying that hm‐CNB adsorbs CO_2_ more readily than CNB. Moreover, the charge density difference of adsorbed CO_2_ (∆q) is calculated. As shown in Figure 8c,d, CO_2_ gains approximately 0 e and ‐0.21 e electrons from CNB and hm‐CNB, respectively, indicating that the heterojunction between hm‐CN and BOB accelerates electron migration and CO_2_ activation.

### 3.4 Photoresponse and Electrochemical Characterization

**Figure 9:**
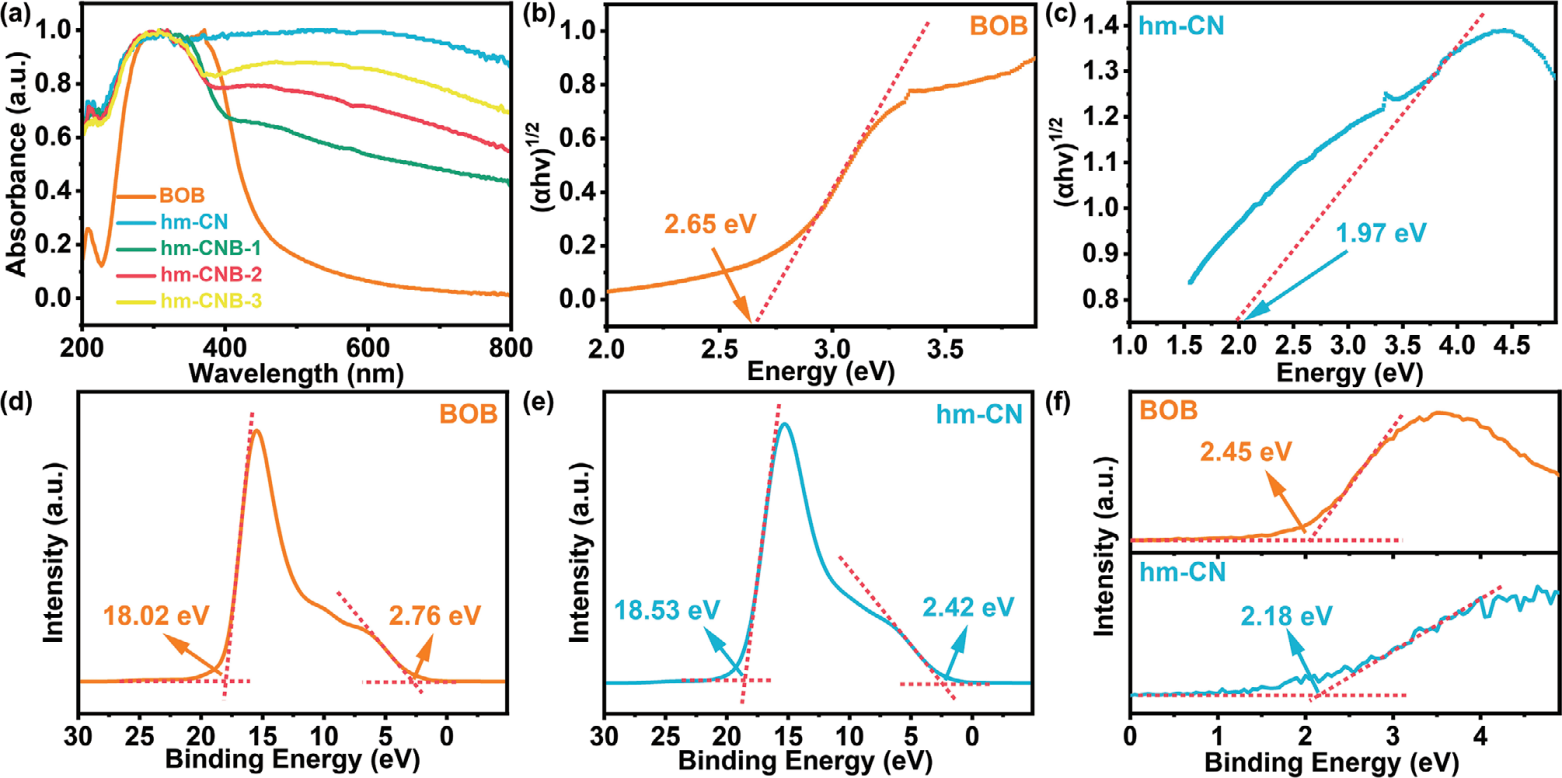
a) DRS spectra of BOB, hm‐CN, and hm‐CNB. b,c) Tauc curves, d,e) UPS spectra, and f) VB‐XPS plots of BOB and hm‐CN.

To elucidate the mechanism for the improved photocatalytic CO_2_ reduction activity, ultraviolet‐visible diffuse reflectance spectroscopy (DRS) is conducted. BOB exhibits strong absorbance in the 200—500 nm range with the maximum absorption edge at 600 nm. In contrast, hm‐CN shows a broader absorption range that extends to 800 nm due to its black appearance. The absorbance of the composite in the ultraviolet and visible light regions increases in spite of a relatively small mass ratio of hm‐CN in hm‐CNB‐1. The hm‐CNB composite exhibits different color from grey to black confirming the strong sunlight trapping ability of hm‐CN (**Figure** **9**a). The greater the concentration of hm‐CN, the stronger the light absorption. Among them, hm‐CNB‐3 exhibits the highest light absorption due to the largest loading capacity of hm‐CN.

The band structures of BOB and hm‐CN are examined. The bandgaps (E_g_) of BOB and hm‐CN are calculated to be 2.65 eV and 1.97 eV by αhυ = A (hυ‐E_g_)^n/2^ (Figure 9b,c), respectively. As a narrow bandgap semiconductor, hm‐CN shares the same half‐metallic characteristics. The work functions of BOB and hm‐CN are determined by ultraviolet photoelectron spectroscopy (UPS, Figure 9d,e) and XPS valence band spectra (VB‐XPS). The work functions are 3.20 eV and 2.69 eV and the valence band maxima (VBM) are at 2.45 eV and 2.18 eV (Figure 9f), respectively, relative to the Fermi level. The positions relative to the vacuum level are determined to be 5.65 eV and 4.87 eV by E_VBM_ = E_VBM‐XPS_ + φ. The conduction band minima (CBM, vs. vacuum level) determined by the formula E_CBM_ = E_VBM_ – E_g_, are 3.0 eV and 2.9 eV for BOB and hm‐CN, respectively.^[^ ^42^, ^43^ ^]^

To investigate the heterojunction type of hm‐CNB as well as the electron transport pathways, the H_2_O oxidation of the material to produce O_2_ was tested. As shown in Figure S9a (Supporting Information), both BOB and hm‐CNB demonstrated photocatalytic oxidation of water for 5 h with O_2_ production, while hm‐CN had no O_2_ yield. According to the oxygen production potential of H_2_O oxidation is ‐5.32 eV (pH = 7),^[^ ^44^ ^]^ it can be seen that the valence band of BOB is more negative, while the valence band of hm‐CN is more positive. Therefore, BOB has the ability to produce O_2_, while hm‐CN cannot. Assuming that the heterojunction formed by the material is a Z‐scheme, the electrons transfer from the VBM of BOB to the CBM, then from the CBM of BOB to the VBM of hm‐CN. The transferred electrons bind with the holes on the hm‐CN, which promotes the transfer of the electrons on the VBM of hm‐CN to the CBM to participate in the CO_2_ reduction reaction (Figure S9b, Supporting Information). Then the position of the effective band involved in the reaction is from the VBM of BOB to the CBM of hm‐CN. The potential of hm‐CNB‐2 involved in the oxidation reaction is ‐5.65 eV, which is lower than the oxygen production potential. Consequently, hm‐CNB‐2 is capable of producing oxygen normally, matching the experimental results. Secondly, assuming hm‐CNB to be a p‐n heterojunction, electrons transfer from the CBM of hm‐CN to the CBM of BOB while holes transfer from the VBM of BOB to the VBM of hm‐CN. However, the effective VBM of hm‐CNB involved in the reaction is ‐4.87 eV and thus incapable of oxidizing H_2_O to produce oxygen, which contradicts the experimental results. Therefore, it can be suggested that the charge migration path of hm‐CNB during photoreaction follows the mechanism of Z‐scheme heterostructure.^[^ ^45^ ^]^

**Figure 10:**
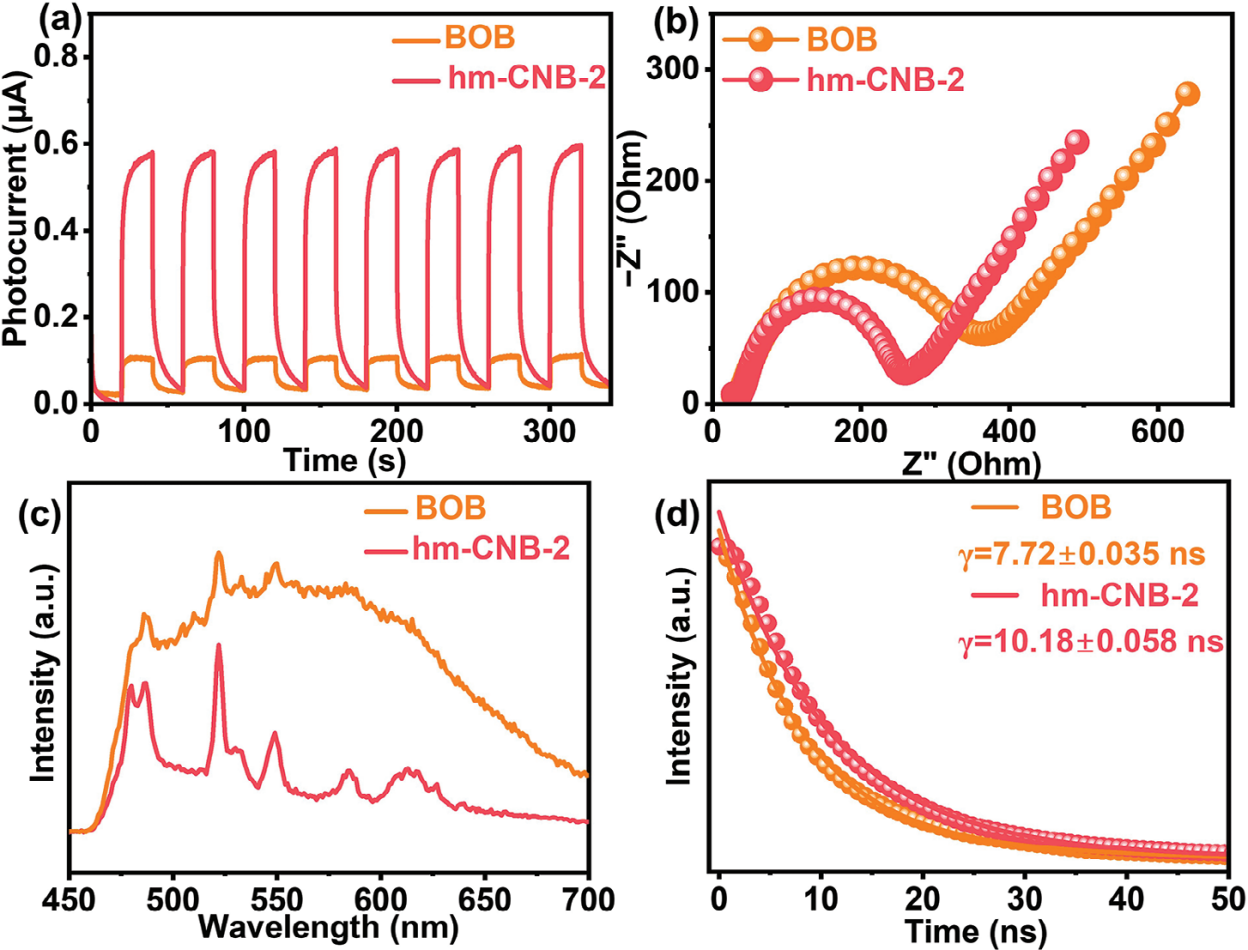
a) Transient photocurrent response, b) EIS spectra, c) PL spectra, and d) Time‐resolved PL spectra of BOB and hm‐CNB‐2.

In order to determine the factors contributing to the variations in the photocatalytic activity among different samples, the photocurrents, electrochemical impedance spectroscopy (EIS), steady photoluminescence (PL), and time‐resolved PL spectroscopy are used to examine the behavior of the light‐induced charge carriers in BOB and hm‐CNB‐2.^[^ ^14^, ^46^, ^47^ ^]^ Transient photocurrent spectroscopy reveals that the photocurrent of BOB is low due to severe recombination of photogenerated electron‐hole pairs. However, after introduction of hm‐CN, the photocurrent of hm‐CNB‐2 increases due to enhanced charge separation (**Figure** **10**a).^[^ ^17^, ^38^ ^]^ In addition, when the hm‐CN concentration in hm‐CNB is small, many BOB particles are not encapsulated. Conversely, when an excessive amount of the half‐metallic material is added, the thicker hm‐CN layer obstructs light trapping by individual BOB particles, thereby affecting the photocatalytic activity. As shown in Figure 6a, an excessive amount of hm‐CN decreases the CO activity because the longer carrier migration path reduces interfacial charge separation and the uneven distribution of hm‐CN undermines the contact between BOB and hm‐CN. The smaller radius in the EIS curve indicates higher interface charge transfer capability (Figure 10b).^[^ ^15^, ^48^, ^49^ ^]^ The resistance of hm‐CNB‐2 is smaller lower than that of BOB, indicating that the Z‐scheme heterojunction formed by the combination of BOB and hm‐CN promotes charge transfer and suppresses carrier recombination. BOB exhibits a PL emission peak at 525 nm which corresponds to band‐to‐band emission (Figure 10c). After combining BOB and hm‐CN, the intensity of hm‐CNB‐2 decreases implying inhibited charge recombination (Figure 10d). Time‐resolved PL spectroscopy also reveals that the electron lifetime of hm‐CNB‐2 is longer than that of BOB. The results verify enhanced charge carrier separation. The longer hole and electron lifetimes and improved charge transfer enhance the photocatalytic activity.

### 3.5 Mechanism of Photocatalytic CO_2_ Reduction

**Figure 11:**
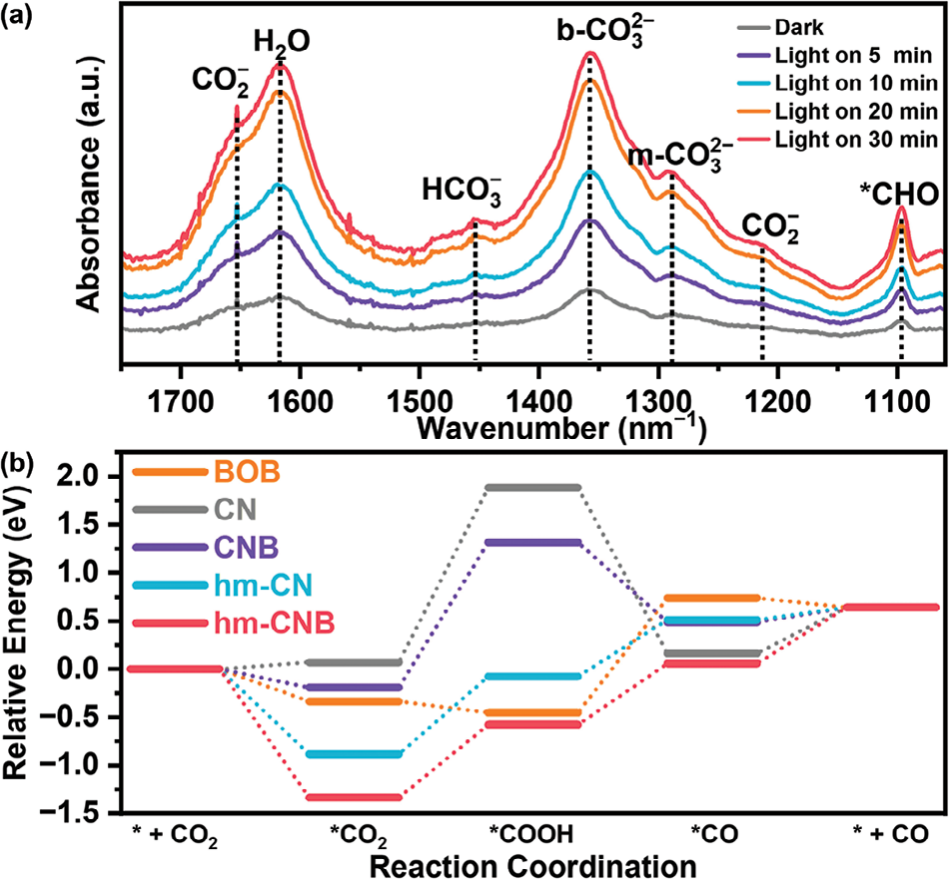
Intermediate processes in CO_2_ photoreduction. a) In situ FTIR spectra for the CO_2_ reaction of hm‐CNB‐2 and b) Free energy diagrams of the catalyst surface.

The CO_2_ absorption and activation processes are studied by in situ Fourier transform infrared spectroscopy (in situ FTIR) as shown in **Figure** **11**a. Using the same testing method described above, various species such as H_2_O (1,617 cm^−1^), HCO_3_^−^ (1,455 cm^−1^), b‐CO_3_^2−^ (1,357 cm^−1^), and *CHO (1,097 cm^−1^) are identified in the dark. After exposing to light for a certain period of time, the peaks at 1,653, 1,358, and 1,097 cm^−1^ corresponding to the intermediates of CO_2_^−^, b‐CO_3_^2−^, and *CHO, respectively, increase.^[^ ^50^, ^51^ ^]^ As the irradiation time goes up, the intensity of the intermediates also increases, suggesting strong electron transfer between BOB and hm‐CN and promoted charge separation and absorption of the intermediate *COOH. Consequently, the reactions for photocatalytic reduction are proposed as shown in the following:

(3) $${\mathrm{CO}}_{2}+*\to *CO_{2}$$

(4) $$*CO_{2}+H^{+}+e^{-}\to *\mathrm{COOH}$$

(5) $$*\mathrm{COOH}+H^{+}+e^{-}\to *\mathrm{CO}+H_{2}O$$

(6) ∗CO→CO↑+∗

(7) $$*CH_{2}O+H^{+}+e^{-}\to *CH_{3}O$$

(8) $$*CH_{2}O+H^{+}+e^{-}\to *CH_{3}O$$

(9) $$*CH_{3}O+3H^{+}+3e^{-}\to CH_{4}↑+H_{2}O+*$$

Figure 11b; Figure S10–S14 and Table S2—S7 (Supporting Information) show that the formation of *CO intermediate on both BOB surfaces is the rate determining step. The key step for CN is the formation of *COOH. When CN forms a complex with BOB, the key step of BOB shifts to *COOH formation. By creating a Z‐scheme heterojunction by combining hm‐CN with BOB, the energy required for hm‐CNB to generate the *COOH intermediates decreases significantly, thereby enhancing the thermodynamic activity during CO_2_ reduction.

By considering the experimental and simulated data, the possible mechanism is postulated. Upon exposure to sunlight, electrons from the VBM in BOB are excited and migrate to the CBM to create electrons and holes. Without hm‐CN, rapid recombination of electrons and holes occurs in the bulk and on the surface of BOB. However, in the presence of hm‐CN, the compact interface forms between BOB and hm‐CN enhances carrier transfer. On account of the energy level difference, electrons from BOB are transferred to hm‐CN and react with CO_2_ molecules adsorbed on the surface of hm‐CN to produce CO. As a result, the heterojunction in hm‐CNB enhances the activity in photocatalytic CO_2_ reduction.

## 4 Conclusion

A novel strategy is designed and demonstrated to synthesize the hm‐CNB composite catalysts for photocatalytic CO_2_ reduction. hm‐CN coverage on BOB produces superior CO_2_ capture efficiency, and the well‐defined heterojunction interfaces expose abundant reaction sites. As a result, hm‐CN has outstanding CO_2_ capture, activation, and charge transfer properties, which in turn improve the photocatalytic CO_2_ conversion efficiency and yield for CO and CH_4_. The optimized hm‐CNB‐2 composite shows outstanding photocatalytic activity because the heterojunction promotes the separation of photoinduced charge carriers and enhances the redox capacity. The results reveal a user‐friendly method for the development and synthesis of photocatalysts composed of hm‐CN and provide insights into the research of artificial photosynthesis and related applications.

## Conflict of Interest

The authors declare no conflict of interest.

## Author Contributions

X.Z. performed methodology, investigation, conceptualization, data curation, wrote ‐the original draft. H.X. performed investigation, wrote ‐the original draft. J.L. performed methodology, wrote – review & edited the original draft. C.B. performed resources, methodology, wrote – review & edited the original draft. J.T., K.Z., B.W. Wrote – review & edited and investigated the original draft. P.D. performed resources, investigation, writing – review & edited the original draft. X.W. performed resources, funding acquisition. H.X. performed methodology, resources, funding acquisition. P.K.C. performed resources, funding acquisition, Wrote – review & edited the original draft. J.D. performed resources, funding acquisition, investigation.

## Supporting information

Supporting Information

## Data Availability

The data that support the findings of this study are available from the corresponding author upon reasonable request.
