# Supplementary material for: Stacking Engineering of Heterojunctions in Half‐Metallic Carbon Nitride for Efficient CO2 Photoreduction
Source: Adv Sci (Weinh). 2023 Dec 10;10(36):2307192. doi: 10.1002/advs.202307192 (PMC10754085; doi:10.1002/advs.202307192)
Supplement: Supplementary file 1 — Supporting Information [file ADVS-10-2307192-s001.pdf]

ADVANCED SCIENCE
Open Access

Supporting Information

for *Adv. Sci.*, DOI 10.1002/advs.202307192

Stacking Engineering of Heterojunctions in Half-Metallic Carbon Nitride for Efficient CO_2 Photoreduction

Xingwang Zhu, Hangmin Xu, Jinyuan Liu, Chuanzhou Bi, Jianfeng Tian, Kang Zhong, Bin Wang, Penghui Ding, Xiaozhi Wang*, Paul K. Chu, Hui Xu* and Jianning Ding**

Supporting Information

Stacking Engineering of Heterojunctions in Half-Metallic Carbon Nitride for Efficient CO_2 Photoreduction

Xingwang Zhu[a], Hangmin Xu[a], Jinyuan Liu[d], Chuanzhou Bi[a], Jianfeng Tian[a], Kang Zhong[b], Bin Wang[b,d], Penghui Ding[c,], Xiaozhi Wang[a,*], Paul K. Chu[d], Hui Xu[b,*] and Jianning Ding[a,*]*

[a] College of Environmental Science and Engineering, Institute of Technology for Carbon Neutralization, Yangzhou University, Yangzhou 225009, P. R. China

[b] School of the Environment and Safety Engineering, Institute for Energy Research, Jiangsu University, Zhenjiang 212013, P. R. China

[c] Department of Science and Technology, Linköping University, Norrköping SE-601 74, Sweden

[d] Department of Physics, Department of Materials Science and Engineering, and Department of Biomedical Engineering, City University of Hong Kong, Tat Chee Avenue, Kowloon, Hong Kong 999077, P. R. China

* Corresponding authors: penghui.ding@liu.se (P. Ding); xzwang@yzu.edu.cn (X. Wang); xh@ujs.edu.cn (H. Xu); dingjn@yzu.edu.cn (J. Ding)

Materials characterization

The samples were analyzed by X-ray diffraction (XRD) on the Bruker D8 diffractometer with Cu Kα radiation (λ = 1.5418 Å) at a rate of 7° min^{-1} and the Raman spectra were obtained on the Thermo Fisher DXR using a laser wavelength of 532 nm. Transmission electron microscopy (TEM) was performed at 200 kV on the FEI Talos F200X G2 (FEI, USA). Field-emission scanning electron microscopy (FE-SEM) and energy-dispersive X-ray spectroscopy were conducted on the JSM-7800F. X-ray photoelectron spectroscopy (XPS) was carried out on the ESCALAB MKII using Al K_{α} radiation. The ultraviolet-visible (UV-vis) diffuse reflectance data were acquired on the Shimadzu UV-3600 plus (Japan) with $BaSO_4$ used as the reflectance standard. The in situ FTIR spectra were obtained on the Thermo Scientific Nicolet iS50 and the photoluminescence (PL) spectra were acquired on the Quanta Master & Time Master Spectrofluorometer.

Photocatalytic activity evaluation

The photocatalytic activity was assessed on the photoreaction system (Labsolar-6A, Beijing Perfectlight). The sample (10 mg) was dissolved in 10 mL of water in a 300 mL Pyrex reaction which was evacuated to a pressure less than 1 kPa before CO_2 was bled in to reach a working pressure of 75 kPa. The reaction proceeded at a low temperature (10°C) irradiated with a 300 W Xenon lamp (PLS-SXE 300C (BF), Beijing Perfectlight). The gas product was analyzed by gas chromatography (GC2002, Ke Chuang, Shanghai).

Photoelectrochemical measurements

The electrochemical impedance spectroscopy (EIS) measurements were conducted on the CHI 660B electrochemical system using the standard three-electrode system. The sample (4 mg) was dispersed in a solution containing 2 mL of ethylene glycol. The suspension (20 μL) was drop-casted onto an ITO-coated glass (0.5 cm × 1 cm) and dried. The working electrode was the ITO substrate with the sample. A Pt wire was the counter electrode and the reference electrode and electrolyte were Ag/AgCl/sat. KCl and Na_2SO_4 (0.2 M).

Theoretical calculation

Density-functional theory (DFT) calculations implemented in the Vienna ab initio Simulation Package (VASP) were performed. The PBE exchange-correlation functional of the generalized gradient approximation (GGA) was used to describe the exchange correlation energy. A plane-wave kinetic-energy cutoff of 520 eV and a maximum force tolerance of 0.05 eV/Å were employed to obtain converging results. A vacuum thickness of 10 Å was adopted in the z-direction to avoid interactions between periodic slab images. The free energy change (ΔG) for adsorption was determined by the following equation:

$$\Delta G = E_{total} - E_{slab} - E_{mol} + \Delta E_{ZPE} - T\Delta S,$$

where E_{total} is the total energy of the adsorption state, E_{slab} is the energy of the pure surface, E_{mol} is the energy of the adsorption substrate, ΔE_{ZPE} is the zero-point energy change, and ΔS is the entropy change.

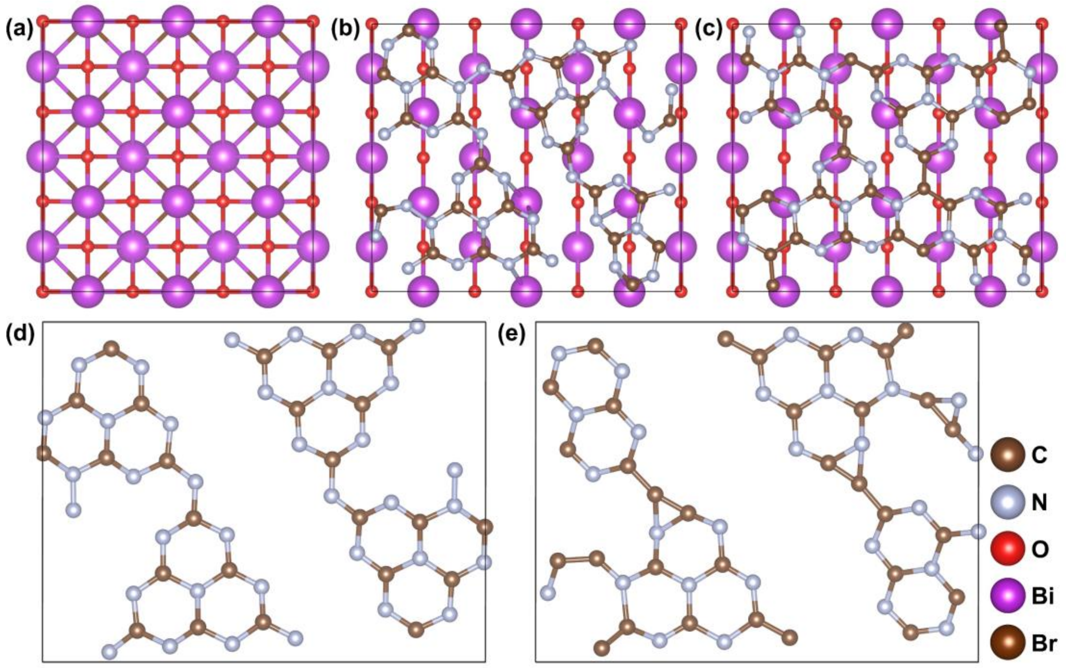

Supplementary Figure S1. Structural models of (a) BOB, (b) CNB, (c) hm-CNB, (d) (e) CN, and (f) hm-CN.

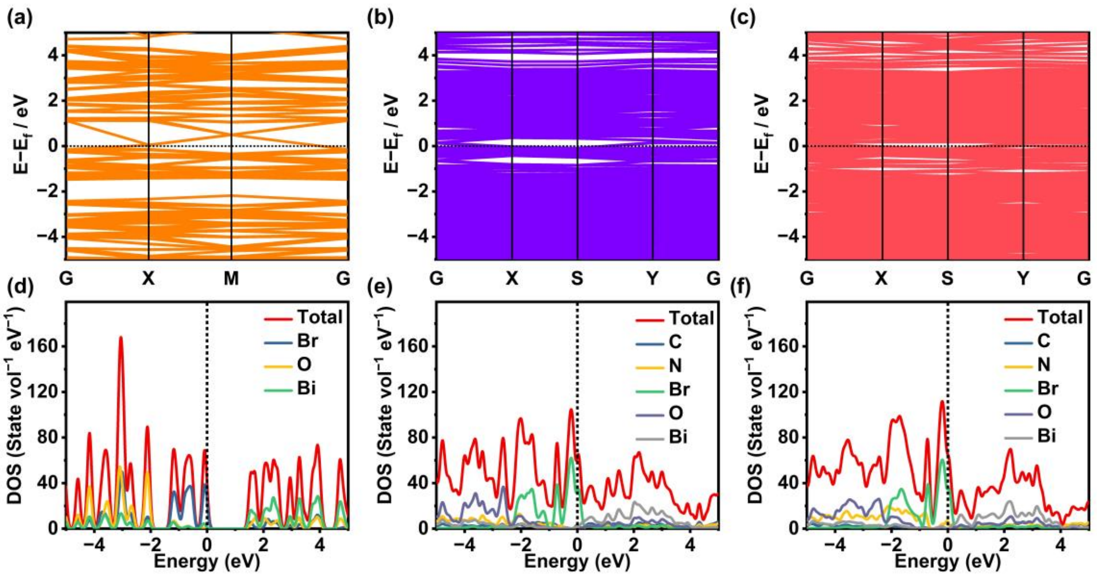

Supplementary Figure S2. Band structures of (a) BOB, (b) CNB, and (c) hm-CNB; DOS of (d) BOB, (e) CNB, and (f) hm-CNB with the dashed lines denoting the Fermi level set at 0 eV.

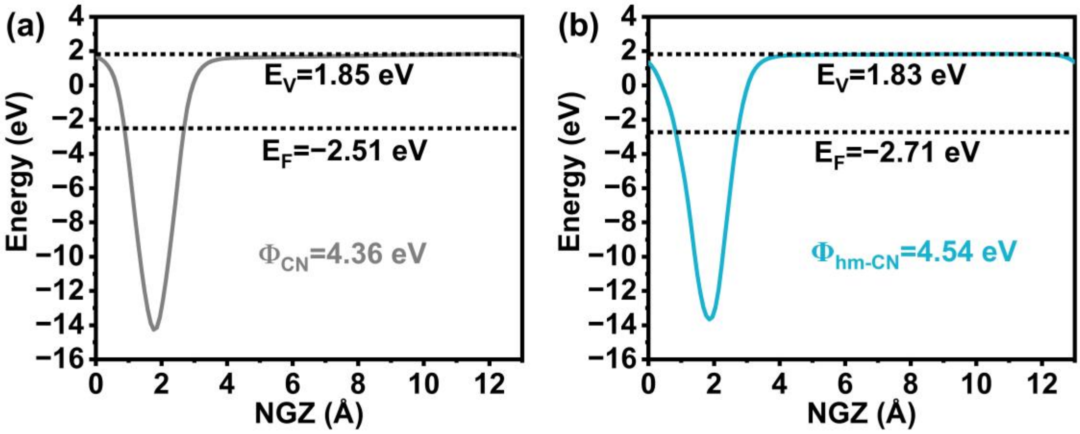

Supplementary Figure S3. Calculated electro-potentials along the z-axis: (a) CN and (b) hm-CN.

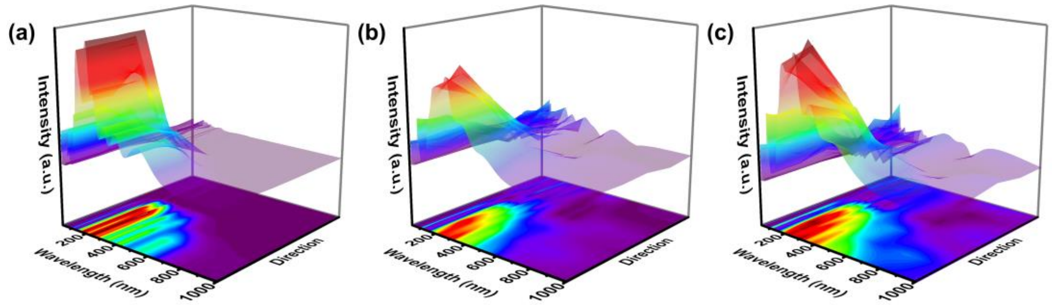

Supplementary Figure S4. Calculated absorption spectra in different directions: (a) BOB, (b) CNB, and (c) hm-CNB.

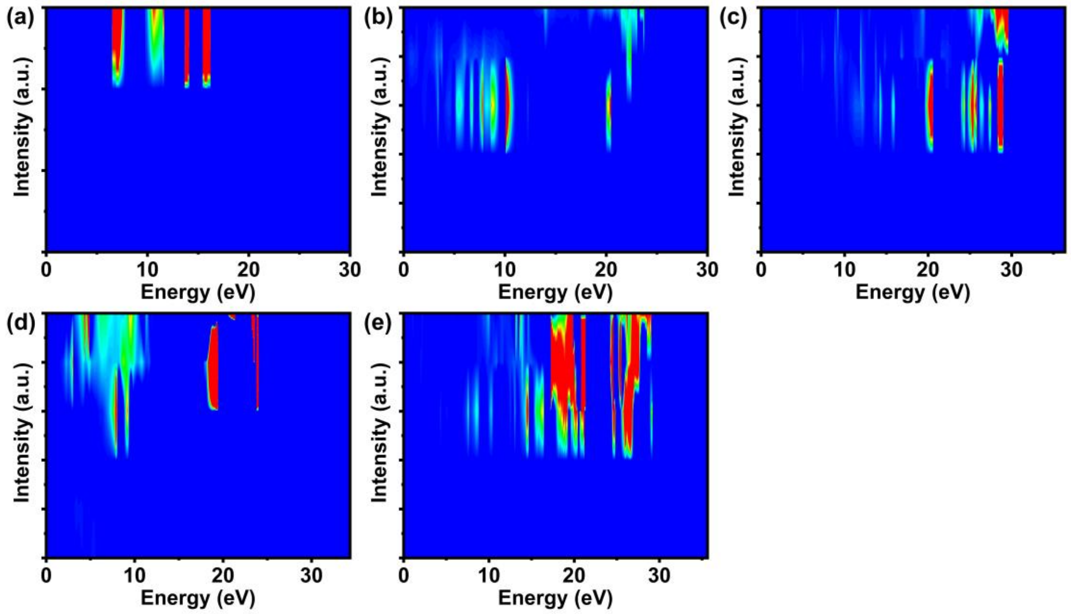

Supplementary Figure S5. Calculated electron energy loss spectra in different directions: (a) CN, (b) hm-CN, (c) BOB, (d) CNB, and (e) hm-CNB.

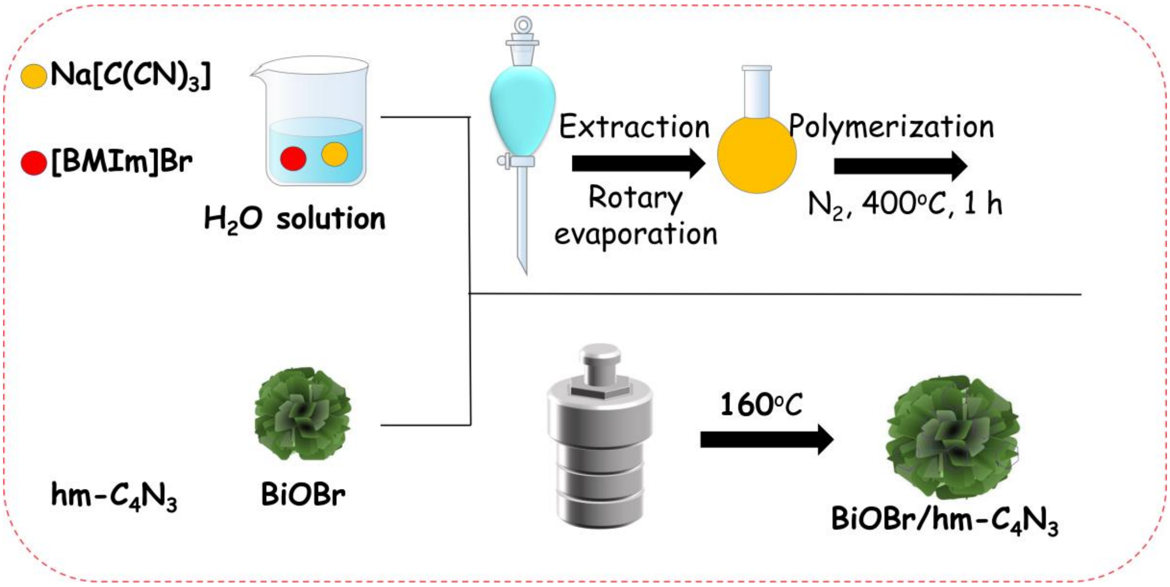

Supplementary Figure S6. Illustration of the sample preparation process.

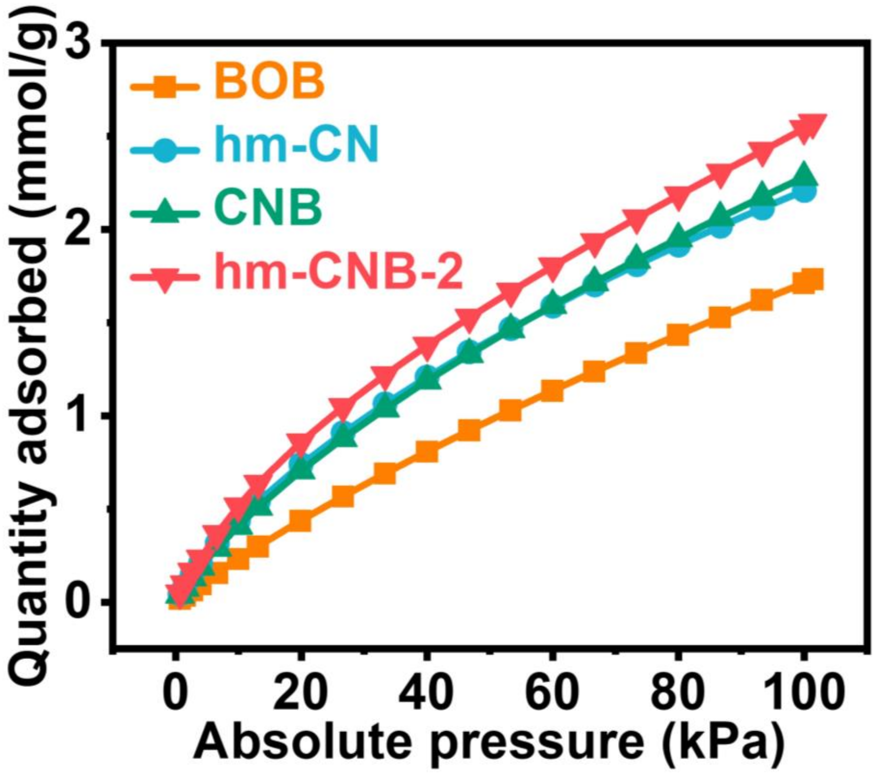

Supplementary Figure S7. CO_2 adsorption performance of BOB, hm-CN, CNB and hm-CNB.

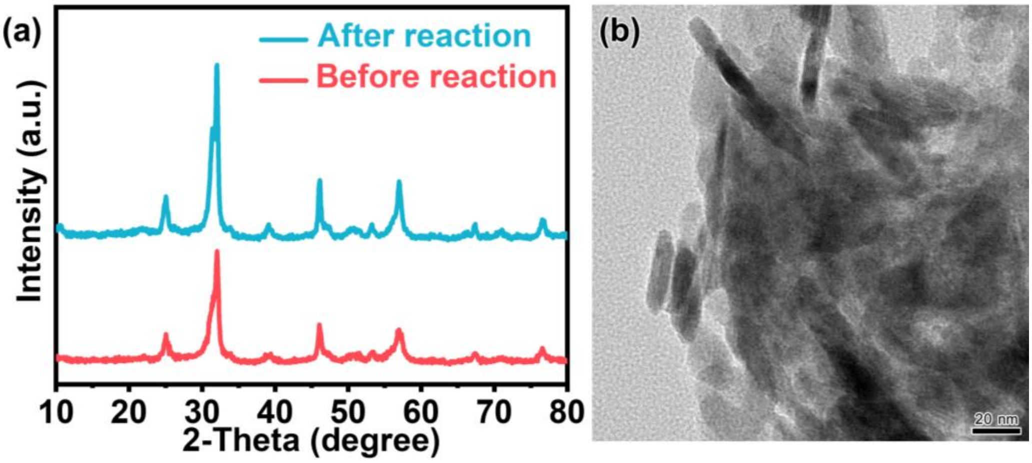

Supplementary Figure S8. (a) XRD patterns of hm-CNB-2 before and after the cycling photocatalytic. (b) TEM image of hm-CNB-2 after the cycling photocatalytic.

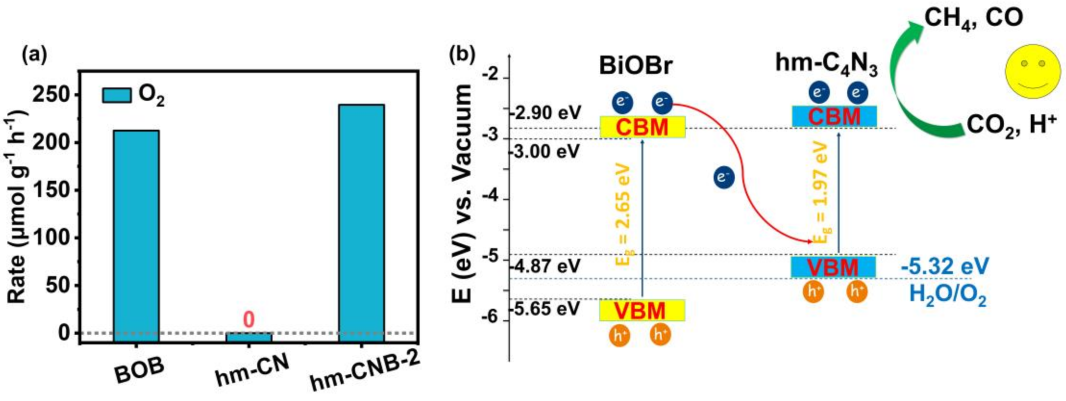

Supplementary Figure S9. (a) Oxidizing H_2O to O_2 performance test of BOB, hm-CN and hm-CNB-2. (b) Band structures of BOB and hm-CN.

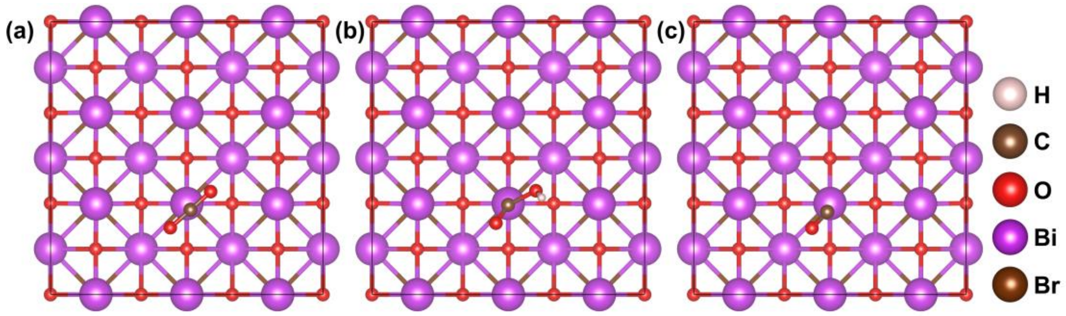

Supplementary Figure S10. Structural models of BOB for free energy calculation.

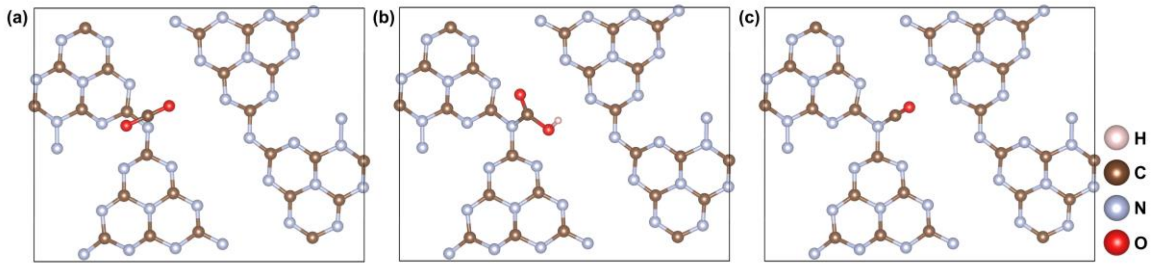

Supplementary Figure S11. Structural models of CN for free energy calculation.

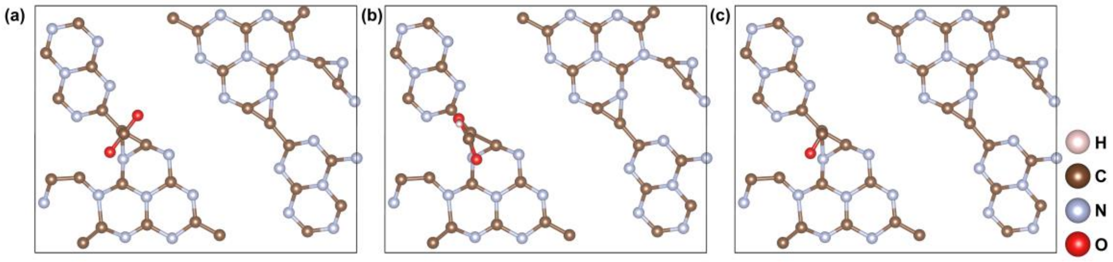

Supplementary Figure S12. Structural models of hm-CN for free energy calculation.

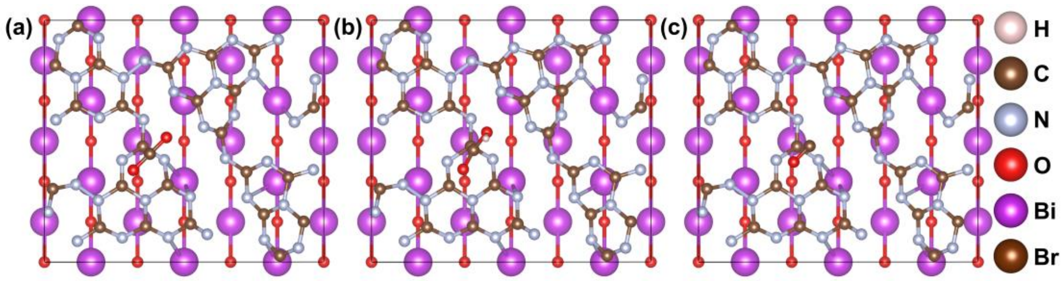

Supplementary Figure S13. Structural models of CNB for free energy calculation.

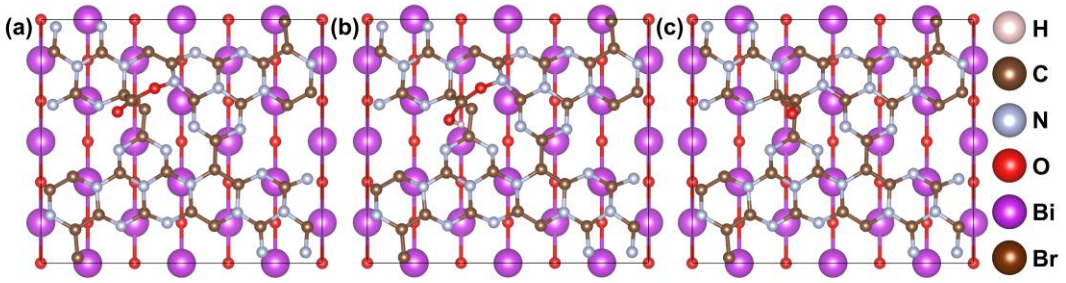

Supplementary Figure S14. Structural models of hm-CNB for free energy calculation.

Supplementary Table S1. Comparison of the CO production rates of different catalysts in photocatalytic CO_2 reduction.

Photocatalyst	Reaction medium	Main products (μmol h^{-1} g^{-1})	Ref.
hm-C_4N_3/BiOBr	300 W Xe-lamp, H_2O, TEOA as sacrificial agent, 10 mg catalyst	183.62 (CO)	**This work**
$BiOV_4$/hm-CN-20	300 W Xe-lamp, H_2O, 100 mg catalyst	40.8 (CO)	[S1]
Co_2N/BiOBr-1	300 W Xe-lamp, H_2O, 30 mg catalyst	67.8 (CO)	[S2]
Oxygen-defect BiOBr atomic layers	300 W Xe-lamp, H_2O, 100 mg catalyst	87.4 (CO)	[S3]
Au_{25} NCs/BiOBr	300 W Xe-lamp, H_2O, CH_3CN, TEOA as sacrificial	43.57 (CO)	[S4]

	agent, 10 mg catalyst		
AgBr/BiOBr	300 W Xe-lamp, H_2O, 10 mg catalyst	212.6 (CO)	[S5]
BiOBr/CdS	300 W Xe-lamp, H_2O, H_2SO_4, 50 mg catalyst	19.4 (CO)	[S6]
Ultrathin BiOBr	300 W Xe-lamp, H_2O, 150 mg catalyst	2.67 (CO)	[S7]

Supplementary Table S2. Energies (eV) of BOB and corresponding groups.

BOB	Slab (*)	*CO_2	*COOH	*CO	CO
E/eV	-242.94	-266.46	-270.39	-258.06	-
ΔE/eV	0.00	-0.54	-0.55	1.49	0.33
ΔG/eV	0.00	-0.34	-0.12	1.19	-0.10
G/eV	0.00	-0.34	-0.45	0.74	0.64

Supplementary Table S3. Energies (eV) of CN and corresponding groups.

CN	Slab (*)	*CO_2	*COOH	*CO	CO
E/eV	-501.09	-524.20	-526.20	-516.79	-
ΔE/eV	0.00	-0.13	1.38	-1.41	0.91
ΔG/eV	0.00	0.07	1.81	-1.72	0.48
G/eV	0.00	0.07	1.88	0.16	0.64

Supplementary Table S4. Energies (eV) of CNB and corresponding groups.

CNB	Slab (*)	*CO_2	*COOH	*CO	CO
E/eV	-729.49	-752.86	-755.17	-744.86	-
ΔE/eV	0.00	-0.39	1.07	-0.52	0.58
ΔG/eV	0.00	-0.19	1.51	-0.83	0.15
G/eV	0.00	-0.19	1.32	0.49	0.64

Supplementary Table S5. Energies (eV) of hm-CN and corresponding groups.

hm-CN	Slab (*)	*CO_2	*COOH	*CO	CO
E/eV	-490.60	-514.67	-517.67	-505.95	-
ΔE/eV	0.00	-1.08	0.37	0.89	0.56
ΔG/eV	0.00	-0.88	0.81	0.59	0.13
G/eV	0.00	-0.88	-0.08	0.51	0.64

Supplementary Table S6. Energies (eV) of hm-CNB and corresponding groups.

hm-CNB	Slab (*)	*CO_2	*COOH	*CO	CO
E/eV	-730.99	-755.50	-758.56	-746.79	-
ΔE/eV	0.00	-1.54	0.32	0.94	1.01
ΔG/eV	0.00	-1.34	0.76	0.64	0.58
G/eV	0.00	-1.34	-0.58	0.06	0.64

Supplementary Table S7. Energy (eV) of different species and zero point energy (ZPE) and TS (T: Temperature, S: Entropy) contributions to the free energies under standard conditions.

Species	E/eV	ZPE/eV	TS/eV
CO_2	-22.98	0.31	0.66
CO	-14.79	0.13	0.60
H_2O	-14.21	0.56	0.67
H_2	-6.76	0.27	0.40
*CO_2	-	0.16	0.31
*COOH	-	0.41	0.19
*CO	-	0.2	0.24

Based on Table S7, ΔZPE and TΔS of CO_2 can be calculated as follows:

$CO_2 + * \rightarrow {}^*CO_2$ TΔS = -0.35 eV; ΔZPE=-0.15 eV (S1)

${}^*CO_2 + H^+ + e^- \rightarrow {}^*COOH$ TΔS = -0.32 eV; ΔZPE=0.115 eV (S2)

${}^*COOH + H^+ + e^- \rightarrow {}^*CO + H_2O$ TΔS = 0.52 eV; ΔZPE=0.215 eV (S3)

${}^*CO \rightarrow CO\uparrow + *$ TΔS = 0.36 eV; ΔZPE=-0.07 eV (S4)

$$\Delta G_{S1} = \Delta G_{*CO_2} - \Delta G_{CO_2} - \Delta G_{*}$$

$$\Delta G_{S2} = \Delta G_{*COOH} - \Delta G_{*CO_2} - \Delta G_{H}$$

$$\Delta G_{S3} = \Delta G_{*CO} + \Delta G_{H_2O} - \Delta G_{*COOH} - \Delta G_{H}$$

$$\Delta G_{S4} = \Delta G_{CO} + \Delta G_{*} - \Delta G_{*CO}$$

References

[S1] J. Wu, L. Xiong, Y. Hu, Y. Yang, X. Zhang, T. Wang, Z. Tang, A. Sun, Y. Zhou, J. Shen, Z. Zou, Organic half-metal derived erythroid-like $BiVO_4$/hm-C_4N_3 Z-Scheme photocatalyst: Reduction sites upgrading and rate-determining step modulation for overall CO_2 and H_2O conversion, *Appl. Catal. B: Environ.*, **2021**, 295: 120277.

[S2] Di, J.; Chen, C.; Zhu, C.; Song, P.; Duan, M.; Xiong, J.; Long, R.; Xu, M.; Kang, L.; Guo, S.; Chen, S.; Chen, H.; Chi, Z.; Weng, Y.-X.; Li, H.; Song, L.; Wu, M.; Yan, Q.; Li, S.; Liu, Z., Cobalt nitride as a novel cocatalyst to boost photocatalytic CO_2 reduction. *Nano Energy*, **2021,** 79, 105429.

[S3] Wu, J.; Li, X.; Shi, W.; Ling, P.; Sun, Y.; Jiao, X.; Gao, S.; Liang, L.; Xu, J.; Yan, W.; Wang, C.; Xie, Y., Efficient Visible-Light-Driven CO_2 Reduction Mediated by Defect-Engineered BiOBr Atomic Layers. *Angew. Chem. Int. Ed.* **2018,** 57, 8719-8723.

[S4] J. Tian, K. Zhong, X. Zhu, J. Yang, Z. Mo, J. Liu, J. Dai, Y. She, Y. Song, H. Li, H. Xu, Highly exposed active sites of Au nanoclusters for photocatalytic CO_2 reduction, *Chem. Eng. J.*, **2023**, 451, 138392.

[S5] Z. Miao, Q. Wang, Y. Zhang, L. Meng, X. Wang, In situ construction of S-scheme AgBr/BiOBr heterojunction with surface oxygen vacancy for boosting photocatalytic CO_2 reduction with H_2O, *Appl. Catal. B: Environ.*, **2022**, 301, 120802.

[S6] Y. Huang, J. Zhang, K. Dai, C. Liang, G. Dawson, Efficient solar-driven CO_2

reduction on aminated 2D/2D BiOBr/CdS-diethylenetriamine S-scheme heterojunction, *Ceram. Int.*, **2022**, 48, 8423-8432.

[S7] Ye, L.; Jin, X.; Liu, C.; Ding, C.; Xie, H.; Chu, K. H.; Wong, P. K., Thickness-ultrathin and bismuth-rich strategies for BiOBr to enhance photoreduction of CO_2 into solar fuels. *Appl. Catal. B: Environ.* **2016,** 187, 281-290.
